# Quality of Life After Intensive Care Unit Discharge in a Tertiary Care Hospital in India: Cost Effectiveness Analyis

**DOI:** 10.5005/jp-journals-10071-23133

**Published:** 2019-03

**Authors:** Shakti Bedanta Mishra, Banani Poddar, Rajesh Kasimahanti, Afzal Azim, Ratender Kumar Singh, Mohan Gurjar, Arvind Kumar Baronia

**Affiliations:** 1 Department of Critical Care Medicine, IMS and SUM Hospital, Bhubaneswar, Odisha, India; 2,4-7 Department of Critical Care Medicine, Sanjay Gandhi Post Graduate Institute of Medical Sciences (SGPGIMS), Lucknow, Uttar Pradesh, India; 3 Department of Critical Care Medicine, Yashoda Hospital, Secunderabad, Telangana, India

**Keywords:** Cost analysis, Quality of life, Survivor

## Abstract

**Background:**

Improvements in intensive care in the last few decades have shifted the focus from mortality to quality of life of survivors as a more important outcome measure. Allocation of public resources towards intensive care is an important challenge for healthcare administrators. This challenge is made more arduous in resource limited countries like India. Thus, it is imperative to consider patient centerd outcomes and resource utilisation to guide allocation of funds. The aim of this study was to evaluate the quality of life of long-term survivors, and to perform cost-effectiveness and cost-utility analysis.

**Methods:**

Data was retrieved from the records and included age, gender, admission diagnosis, length of ICU stay and mortality. Costing methodology used was top down approach. Quality of life was assessed by SF 36 scoring which was done with personal interview and telephonically. Cost-effectiveness analysis was done on the basis of years of life added. Cost utility was done by QALY gained.

**Results:**

A total of 1232 adult patients were admitted in the period with 758 (61%) being successfully discharged from ICU with a mortality rate of 39%. Out of 758, we could contact 113 (15%) patients. 86 patients were alive at the time of contact who could fill the forms for quality of life. The patients discharged from ICU had scores almost similar to the general population. Lesser scores were noted in physical functioning and general health perceptions, though this difference was not statistically significant. The life years gained were significantly more in younger patients. The cost per life gained was more in patients aged more than 50 years compared to those who were younger.

**Conclusion:**

The quality of life after survival from ICU is as good as in the general population. The intensive care provided in our ICU is cost effective.

**How to cite this article:**

Mishra SB, Poddar B et al, Quality of Life After Intensive Care Unit Discharge in a Tertiary Care Hospital in India: Cost Effectiveness Analysis. Indian J Crit Care Med 2019;23(3):122-126.

## INTRODUCTION

Mortality is one of the most important outcome parameters measured in the intensive care unit (ICU)^1,2^. Improvements in intensive care in the last few decades have shifted the focus from mortality to quality of life of survivors as a more important outcome measure^3^. There have been numerous studies from developed nations on the quality of life following survival after ICU stay; similar studies from developing countries like India are scarce^4–6^.

Further, since intensive care utilises a large amount of resources for a limited number of patients, the need to know how intensive care therapy has influenced the quality of life of survivors becomes very important. Allocation of public resources towards intensive care is an important challenge for health care administrators. This challenge is made more arduous in resource limited countries like India. Thus, it is imperative to consider patient centred outcomes and resource utilisation to guide allocation of funds.

The aim of this study was to evaluate the quality of life of long-term survivors, and to perform cost-effectiveness and cost-utility analysis.

### Patients and Methods

The study protocol was approved by the Institute ethics committee of the Sanjay Gandhi Post Graduate Institute of Medical Sciences, Lucknow, India. Consecutive patients who stayed more than 24 hours in the ICU of the Department of Critical Care Medicine between June 2005 and May 2010 were eligible for study entry. Data retrieved included age, gender, admission diagnosis, length of ICU stay and mortality. Patients who died within 2 years of discharge from ICU were termed as non survivors and were not included in our analysis. Costing methodology used was top down approach. Here, the cost that was borne by each patient was included as the cost of therapy. The cost was of the ICU stay duration only and not of the complete hospital stay.

#### Quality of Life and Health Status Index

We extracted patients' contact information like telephone numbers and address from our database. We tried to contact them telephonically and sent letters to their addresses. Patients who came to the hospital for follow up in other departments were interviewed personally and telephonic interviews were done, if required. If patients did not come to the hospital and/or telephone contact could not be established, then a formal letter was sent accompanied by a return envelope and the validated Hindi/English interview form of the Medical Outcome Survey Short Form-36 questionnaire (SF-36)^7,8^ self-report form. QOL was assessed by means of the Medical Outcomes Study 36-item Short Form Health Survey (SF-36v2®). The SF-36 questionnaire contains 36 items measuring eight health domains: physical (PF) and social functioning (SF), role limitations due to physical (RP) or emotional problems (RE), mental health (MH), vitality (VT), bodily pain (BP), and general perception of health (GH). Two component scores physical (PCS) and mental (MCS), are calculated summary scores where the physical domains (PF, RP, BP, GH) or the mental domains (VT, SF, RE, MH) will account more in the score. For a few patients, the same form was sent on email and the completed form was returned to us. Death of the patient was confirmed from relatives wherever this had occurred.

Based on the SF-36, a health status index was calculated for each patient. A health status index assigns a weight ranging from 0 (in difference between life and death) to 1 (perfect health) corresponding to the overall quality of life. In this study, the proportion of the SF-36 in each of the eight domains was set against the measures of an age-matched group of apparently healthy subjects.

#### Cost-Effectiveness and Cost Utility

The comparison of cost-effectiveness analysis with a comparator group requires similar treatment strategy or drugs. Since this was an observational study, no comparator group was available. The results are therefore based on the assumption that patients would have died without ICU care. To estimate the cost per life year gained, the costs per survivor were divided by the estimated life years gained. The remaining life span of the patients was calculated based on an average age-adjusted life expectancy of 64.3 yrs. for males and yrs. for females^9,10^.

Cost utility analysis is a cost-effectiveness analysis in which the quality of life of the patient is utilised to compare the associated cost. It is usually estimated in terms of life years gained and QALY (Quality adjusted Life Years)^11^. A QALY is the product of the number of years of life times the health status index. Costs per QALY were calculated according to the health status index of the patients surviving^12^.

*Statistical Analysis:* All variables were tested for normal distribution with the Kolmogorov-Smirnov test. Descriptive statistics include mean and SD values except when stated otherwise. Student's t-test was used to compare means of continuous normally distributed data. A nonparametric rank test (Mann-Whitney U test) was applied in case of non-normally distributed data. Categorical data were tested using the Chi-square statistic with Yates correction when appropriate. Risk factors were dichotomised to do the cost and risk analysis.

All statistical tests are two-sided, and a significance level of p <0.05 or less was applied, except when stated otherwise. Data were analysed using SPSS 21.0 (SPSS, Chicago, IL).

## RESULTS

The flow chart of patients included in this study is depicted in Flowchart 1. Table 1 illustrates the demographic characteristics of all the patients admitted to the ICU during the period of June 2005 to May 2010. A total 1232 adult patients were admitted in the period with 758 (61%) being successfully discharged from ICU with a mortality rate of 39%. Out of 758, we could contact 113 (15%) patients. 86 patients were alive at the time of contact who could fill the forms for quality of life.

Graph 2 shows the quality of life as evaluated through SF-36 in comparison to the normal general population in a spider diagram. The scoring is divided into 8 parts i.e. vitality (VT), physical functioning (PF), bodily pain (BP), general health perceptions (GH), physical role functioning (RP), emotional role functioning (RE), social role functioning (SF) and mental health (MH). The patients discharged from ICU had scores almost similar to the general population. Lesser scores were noted in physical functioning and general health perceptions, though this difference was not statistically significant.

**Flowchart 1:**
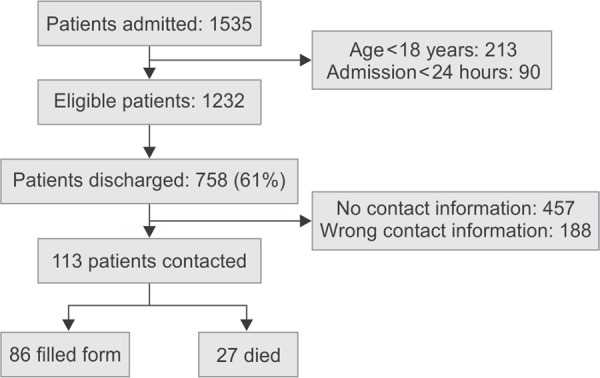
Flow diagram showing how the patients were included

**Table 1:** Demographic profile of all patients

	*All patients*	*Discharged*	*Contacted*
Number	1232	758	113
M:F	818 (66) :414 (34)	529 (70) :229 (30)	70 (62): 43 (38)
Age (mean ± sd)	48.4 ± 16.9	48.5 ± 16.8	44.1 ± 17.1
Diagnosis Tropical Illness	236 (19.1%)	162 (21.3%)	16 (14.2%)
SAP	289 (23.4%)	118 (15.5%)	25 (22.1%)
Neurological	152 (12.3%)	101 (13.3%)	15 (13.3%)
Sepsis	402 (32.6%)	285 (37.5%)	35 (31.0%)
Liver	86 (6.9%)	37 (4.8%)	4 (3.5%)
Others	67 (5.4%)	55 (7.2%)	18 (15.9%)

All values are in number (percentages) else specified. SD: Standard deviation. M: Male. F: Female. SAP: severe acute pancreatitis.

**Graph 1:**
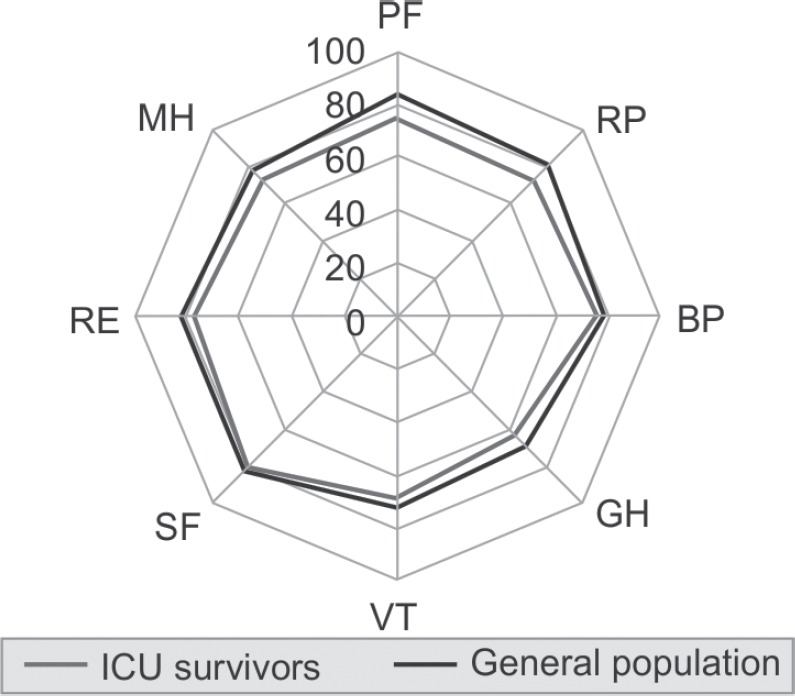
Quality of life as evaluated through SF-36 in comparison to the normal general population

The patients were dichotomised based on age (>50 years and ≤ 50years), duration of mechanical ventilation (>7 days and ≤7days) Cost per life years gained (US$) and stay in the ICU (>28 days and ≤ 28 days). In older patients, who were older the physical functionality was worse. The patients who spent more time on mechanical ventilation had worse vitality and the patients who spent more time in the ICU had more bodily pain (Tables 2 to 4).

**Table 2:** SF 36 scores based on days of mechanical ventilation (MV)

	*MV > 7days*	*MV = 7days*	*P value*
PF^1^	75.1 ± 22.4	73.9 ± 21.6	0.8
RP^2^	75.7 ± 23.1	70.0 ± 23.7	0.27
BP^3^	77.8 ± 26.3	79.0 ± 22.7	0.25
GH^4^	60.9 ± 15.3	66.1 ± 13.8	0.10
VT^5^	64.9 ± 18.2	74.2 ± 15.6	0.01
SF^6^	81.9 ± 20.9	76.6 ± 23.2	0.46
RE^7^	79.0 ± 23.8	74.0 ± 22.1	0.26
MH^8^	71.1 ± 15.9	74.4 ± 15.7	0.34
PCS^9^	49.2 ± 7.5	49.9 ± 7.1	0.65
MCS^10^	49.9 ± 7.6	50.4 ± 7.2	0.73

**Table 3:** SF 36 scores based on length of stay (LOS) in ICU

	*LOS > 28 days*	*LOS = 28 days*	*p value*
PF^1^	79.6 ± 22.6	75.1 ± 21.0	0.91
RP^2^	70.9 ± 25.5	77.1 ± 19.4	0.24
BP^3^	72.1 ± 27.2	83.0 ± 18.0	0.04
GH^4^	62.0 ± 17.0	66.5 ± 9.3	0.17
VT^5^	67.5 ± 18.5	73.0 ± 15.0	0.16
SF^6^	79.1 ± 23.0	82.8 ± 18.7	0.45
RE^7^	79.3 ± 25.3	81.7 ± 18.0	0.15
MH^8^	72.2 ± 16.4	74.5 ± 14.8	0.51
PCS^9^	48.9 ± 7.8	50.9 ± 6.3	0.22
MCS^10^	49.5 ± 7.9	51.6 ± 6.5	0.19

The cost analysis was done based on age dichotomization (Table 5). Patients were expected to die if they did not receive ICU admission. The QALY based on SF 6D was similar between the two groups. The cost per QALY gained was statistically similar between the two groups. The life years gained was calculated from data of WHO for India for the year 2015, i.e., average age-adjusted life expectancy of 64.3 yrs for males and 66.4 yrs for females. The life years gained were significantly more in younger patients. The cost per life gained was more in patients aged more than 50 years compared to those who were younger.

**Table 4:** SF 36 scores based on age at time of admission

	*Age > 50 yrs*	*Age = 50 yrs*	*p value*
PF^1^	61.6 ± 25.4	78.1 ± 19.7	<0.01
RP^2^	64.9 ± 28.0	75.4 ± 21.4	0.09
BP^3^	67.1 ± 24.9	78.5 ± 24.0	0.08
GH^4^	65.9 ± 17.2	63.1 ± 14.1	0.46
VT^5^	70.5 ± 16.4	69.3 ± 17.8	0.81
SF^6^	75.6 ± 23.0	81.8 ± 20.1	0.28
RE^7^	68.9 ± 31.3	79.2 ± 20.0	0.28
MH^8^	69.4 ± 14.4	74.2 ± 16.1	0.09
PCS^9^	45.9 ± 7.3	52.1 ± 8.3	0.27
MCS^10^	47.4 ± 7.1	52.1 ± 7.5	0.24

^1^Physical functioning (PF)

^2^Physical role functioning (RP)

^3^Bodily pain (BP)

^4^General health perceptions (GH)

^5^Vitality (VT)

^6^Social role functioning (SF)

^7^Emotional role functioning (RE)

^8^Mental health (MH)

^9^Physical component score (PCS)

^10^Mental component score (MCS)

**Table 5:** Cost utility analysis

	*Age > 50 yrs*	*Age = 50 yrs*	*p value*
QALY gained	5.3 ± 0.8	5.6 ± 0.7	0.23
Cost per QALY gained (US$)	1413 ± 65.8	1319.6 ± 61.3	0.93
Life years gained	6.0 ± 5.7	32.7 ± 7.9	<0.01
Cost per life years gained (US$)	196.1 ± 89.1	16.9 ± 14.1	<0.01

All Values are in mean ± standard deviation unless specified. QALY: Quality Adjusted Life Years. US$: United States Dollars.

## DISCUSSION

In this follow up study of medical ICU patients, we found a quality of life of ICU survivors comparable to the general population. Further, the cost of ICU care provided seems reasonable.

The time course of physical and mental recovery after ICU stay is a highly individual process, and marked differences have been described in the literature. We have based our study on the recommendations of ESICM^13^. Thus, the long follow up in this study has perhaps been a) selective, to exclude patients who may have deteriorated early after their ICU stay; and b) sufficiently long to allow regaining of the best achievable quality of life.

To compile a rigorous cost-effectiveness analysis is a demanding task. There are many methodological intricacies to consider within the study, and the analysis is likely to be highly reliant on the availability and quality of both cost and outcome assessment. To date, our data represents the longest follow up study of ICU patients including cost-effectiveness and cost-utility analysis from India.

### Demographics

The most common cause of admission in our ICU was sepsis followed by severe acute pancreatitis. Survival in ICU was better in patients who were admitted with tropical illness and sepsis compared to other causes. Severe acute pancreatitis patients had comparably higher mortality. As survivors of pancreatitis were on follow up in the gastro-surgery department of our Institute, patients with this diagnosis are over represented in our follow up study. Survivors of tropical illness are fewer in number as these patients had an acute illness from which they had recovered completely and hence, did not need long term follow up.

We used data for the general population of Australia for comparison of quality of life as comparable data for India was not available^14,15^. The data from our study shows comparable results from ICU survivors compared to the general population. The values were slightly less in terms of physical functioning and general health perceptions which contrast with data from developed nations where the emotional component is more affected. Possibly, since our patients are younger, they are more interested in their physical functioning as their income is dependent on it.

Kaarlola et al.16 in a group of critically ill patients from Finland found much slower improvement in emotional parameters following ICU discharge. Flaatten et al.^17^ found similar quality of life 2 years after ICU discharge compared to general population. Winters et al.^18^ in a systematic review of all the quality of life related outcomes from septic ICU patients concluded that there is a need to look beyond 28 days mortality and into long term quality of life related outcomes. Pettilä et al.^19^ showed lower improvements in vitality and emotional parameters 1 year post ICU discharge.

We dichotomised our patients based on age, duration of mechanical ventilation and duration of stay in the ICU. We compared the quality of life parameters measured using the SF-36 form between the groups based on the above classification. Patients who were older than 50 years had a worse physical role function compared to younger patients. This could probably be because of weaker muscles and higher incidence of neurological problems in older age group. The patients who had more than 7 days stay on the ventilator had worse vitality, probably because these patients had weaker respiratory and cardiovascular function compared to those who could be weaned earlier. The patients who had more than 28 days' stay in ICU had more pain which was presumably due to residual neuromuscular weakness, need for invasive tubes and nutritional deficiencies.

The QALY was calculated from the SF-6D scorings^20^. The effective way of estimating the cost effectiveness of any therapy is by measuring the cost per QALY gained. In the setting of developed nations, US$ 50,000 per QALY is a generally accepted upper limit. This cannot be a bench mark for a resource limited country like India. We estimate per capital GDP to be a bench mark for estimating this upper limit. This is roughly US$ 50,000 for United States of America while for India, it is US$ 1800 as per 2016 International Monetary Fund calculations. The cost per QALY gained in our study was US$ 1396. If we consider US$ 1800 as an upper limit to cost effective therapy in India, then the ICU care provided in our ICU may be considered cost effective.

The other concern is effect of age on post ICU outcomes and its cost effectiveness. Limitation of ICU beds and the cost associated sometimes makes the decision difficult when triaging of old patients is done. We divided our patient into two groups; more than 50 years and less than or equal to 50 years for the age related cost analysis. The cost per QALY was similar between the two groups: US$ 1391 for younger patients and US$ 1413 for older patients. This value was not statistically significant, but since the numbers in our study are small, we may get different results in larger studies. The life years gained was based on survival data from WHO for Indian population. The cost per life year gained was significantly more in older population. Inspite of this, the therapy was cost effective in older patients too.

### Limitations

Our study has various limitations, the most important being the large number of survivors whom we could not contact. This is likely to introduce a bias, as those with whom we could establish contact could be different from the rest of the survivors. It is possible that the survivors whom we could contact were more concerned about their health. This factor may impact the quality of life. The age related data is from a very small population. Cost calculations only include the cost of ICU care and not the entire hospital stay. We believe that the results from our study may only be an indicator. These results need to be confirmed from larger and preferably, multicentric studies.

## CONCLUSION

The quality of life after survival from ICU is as good as in the general population. The intensive care provided in our ICU is cost effective.
